# A Novel Blood Index-Based Model to Predict Hepatitis B Virus-Associated Hepatocellular Carcinoma Recurrence After Curative Hepatectomy: Guidance on Adjuvant Transcatheter Arterial Chemoembolization Choice

**DOI:** 10.3389/fonc.2021.755235

**Published:** 2021-12-24

**Authors:** Yiping Zou, Zhihong Chen, Qi Lou, Hongwei Han, Yuanpeng Zhang, Zhenrong Chen, Zuyi Ma, Ning Shi, Haosheng Jin

**Affiliations:** ^1^ Department of General Surgery, Guangdong Provincial People’s Hospital, Guangdong Academy of Medical Sciences, Guangzhou, China; ^2^ College of Medicine, Shantou University, Shantou, China; ^3^ The Second School of Clinical Medicine, Southern Medical University, Guangzhou, China

**Keywords:** hepatocellular carcinoma, blood index signature, nomogram, recurrence-free survival, adjuvant transcatheter arterial chemoembolization

## Abstract

**Background:**

Postoperative recurrence is a significant obstacle in hepatocellular carcinoma (HCC) treatment. This study aimed to construct a blood index-based model to predict hepatitis B virus-associated HCC (HBV-HCC) recurrence after curative hepatectomy.

**Methods:**

A total of 370 patients who received initially curative hepatectomy for HBV-HCC were included in this study. A novel blood index signature (BIS) was identified and systematically analyzed for its recurrence predictive value. Following this, multivariate Cox regression analysis was performed to build a blood index-based nomogram.

**Results:**

A BIS based on the aminotransferase-to-platelet ratio index and a systemic inflammatory response index was used to construct a nomogram. The model showed good clinical applicability and reliability. Notably, the patients in the high recurrence risk group tended to benefit from adjuvant transcatheter arterial chemoembolization (TACE).

**Conclusion:**

A reliable model was constructed to predict the HBV-HCC recurrence after curative hepatectomy. This model can guide the surgeons in selecting patients with high recurrence risk patients who may benefit from adjuvant TACE.

## Introduction

Hepatocellular carcinoma (HCC), the fourth common cause of cancer worldwide, causes more than 600,000 deaths annually. Hepatitis B virus (HBV) infection is the major HCC contributor worldwide (1, 2). Regarding HCC treatment, only curative resection allows patients with HCC to achieve long-term survival. However, the postoperative high recurrence rate is a significant obstacle in cancer management (3, 4). Even for patients with early-stage with small tumors, the 5-year recurrence rate after surgery is approximately 70% (5).

Currently, transcatheter arterial chemoembolization (TACE) is the standard mainstay of treatment for intermediate-stage HCC (6). Although several studies have reported on adjuvant therapeutic modalities, the role of adjuvant TACE in resectable HCC remains controversial (7–9), which can be attributed to the considerable heterogeneity of HCC. The adjuvant therapy may improve the survival benefits of high-risk patients; however, these benefits may be impaired in low-risk patients. Therefore, identifying novel biomarkers and constructing an accurate prediction model based on postoperative HCC recurrence provide physicians with more appropriate therapeutic options. For example, peripheral blood microRNAs were identified as prognostic predictors in patients with HCC who received TACE (10). The result showed that miR-21 and miR-122 were prognostic biomarkers in HCC patients treated with TACE and correlated with hypoxia-inducible factor-1α (HIF-1α) serum levels.

Obtaining the peripheral blood index is convenient and inexpensive. Previous studies have reported that blood indices such as the neutrophil-to-lymphocyte ratio (NLR), platelet-to-lymphocyte ratio (PLR), monocyte-to-lymphocyte ratio (MLR), aminotransferase-to-platelet ratio index (APRI), aspartate aminotransferase-to-neutrophil ratio index (ANRI), systemic immune-inflammatory index (SII), systemic inflammatory response index (SIRI), fibrinogen-to-albumin ratio (FAR), fibrinogen-to-lymphocyte ratio (FLR), γ-glutamyl transpeptidase-to-platelet ratio (GPR), model for end-stage liver disease (MELD), Albumin-Bilirubin (ALBI) Grade, and prognostic nutritional index (PNI) reflect the HCC survival (11–20). Therefore, this study aimed to construct a novel blood index-based model that can accurately predict HBV-associated HCC (HBV-HCC) recurrence.

## Methods

### Study Population

The data of 370 patients pathologically diagnosed with HBV-HCC who received curative hepatectomy as the first line of treatment were retrospectively collected at the Guangdong Provincial People’s Hospital from January 2013 to December 2019. The exclusion criteria included the following: 1) patients younger than 18 years; 2) patients without the negative surgical margin; 3) patients who underwent neoadjuvant downstage therapy; 4) patients with recurrent HCC; 5) patients with preoperative incomplete data on blood indices; and 6) patients with a follow-up period of less than 6 months. This study was approved by the ethics committee of Guangdong Provincial People’s Hospital (KYZ202132501) and performed following the Declaration of Helsinki.

### Management Protocol

Preoperative blood indices and clinicopathological data within 7 days before surgery were collected retrospectively from the electronic medical record system. Contrast-enhanced CT or contrast-enhanced MRI combined with serum alpha-fetoprotein (AFP) was performed during the first month of the first-year follow-up and every 3 months after that to detect early recurrence. Following this, the patients were recommended to undergo imaging examinations every 6 months for 1 year after surgery. Additionally, imaging examinations were performed if patients had chief complaints such as abdominal pain.

Moreover, a multidisciplinary treatment discussion was conducted for every patient before hepatectomy. As the commonly used adjuvant management, all patients were recommended to receive adjuvant TACE 1–2 months after surgery. The highly selective conventional TACE was used for this adjuvant management.

Recurrence-free survival (RFS) was defined as the time from curative hepatectomy to tumor recurrence at any site. The overall survival (OS) was defined as the time from the curative hepatectomy to death, due to any cause, or last contact.

### Statistical Analyses

Continuous variables were presented as the mean and SD, whereas categorical variables were expressed as frequency and percentage. Univariate Cox regression analysis was performed to estimate the indices associated with RFS, and stepwise multivariate Cox regression analysis was performed to build a blood index signature (BIS). The following formula was used to calculate the BIS: H0 * Exp[Ʃ(βi × xi)]. Furthermore, the univariate and multivariate Cox regression analyses were used to assess the independence of novel signature in evaluating RFS based on clinicopathological variables and construct a predictive nomogram. The area under the receiver operating characteristic (ROC) curve was used to evaluate the predictive performance. Calibration curves were used to compare the association between the actual outcomes and predicted probabilities. The best cutoff value for risk stratification was calculated using the X-tile software (Yale School of Medicine, USA). Additionally, the Kaplan–Meier survival curves with log-rang text and subgroup Cox regression analyses were plotted to compare differences in RFS or OS. Decision curve analysis (DCA) was used to evaluate the clinical applicability of the nomogram.

Statistical analyses in this study were performed using the R software version 4.0.5. Unless otherwise stipulated, a two-tailed p-value <0.05 was considered statistically significant.

## Results

### Patient Characteristics

The clinicopathological characteristics of the study’s cohort are summarized in **Table 1**. The patients were predominantly male, with a solitary tumor, without capsule and microvascular invasions (MVIs), and had well-reserved liver function (Child–Pugh class A). The average age of the cohort is 54.22 years. A total of 141 (38.1%) patients underwent laparoscopic surgery, and 126 (34.1%) received adjuvant TACE. The average tumor size is 5.44 cm. A total of 164 (44.3%) patients with Ki67 ≥ 20% and 172 (46.5%) patients with grade III/IV tumor differentiation were observed. Notably, 171 (46.2%) patients showed negative AFP (AFP < 20 ng/ml) result.

**Table 1:** Baseline patient demographics and preoperative characteristics.

Variables	Group (370)
Age (years)	54.22 ± 11.39
Size (cm)	5.44 ± 3.46
Gender	
Female	45 (12.2)
Male	325 (87.8)
Ki67	
<20%	206 (55.7)
≥20%	164 (44.3)
Multiple tumors	
No	319 (86.2)
Yes	51 (13.8)
Capsule invasion	
No	320 (86.5)
Yes	50 (13.5)
Grade	
I/II	172 (46.5)
III/IV	198 (53.5)
MVI	
No	261 (70.5)
Yes	109 (29.5)
Adjunct TACE	
No	244 (65.9)
Yes	126 (34.1)
AFP (ng/ml)	
<20	171 (46.2)
≥20	199 (53.8)
Laparoscopic surgery	
No	229 (61.9)
Yes	141 (38.1)
Child–Pugh classification	
Class A	343 (92.7)
Class B	27 (7.3)
NLR	2.11 ± 1.09
PLR	116.47 ± 66.09
MLR	0.33 ± 0.16
FAR	0.12 ± 0.49
GPR	0.51 ± 0.62
APRI	0.82 ± 0.78
PNI	37.94 ± 5.01
ALBI	−2.44 ± 0.44
FLR	2.78 ± 10.86
SII	415.48 ± 321.12
SIRI	1.17 ± 0.88
ANRI	15.79 ± 15.38
MELD	5.92 ± 0.35

MVI, microvascular invasion; TACE, transcatheter arterial chemoembolization; NLR, neutrophil-to-lymphocyte ratio; PLR, platelet-to-lymphocyte ratio; MLR, monocyte-to-lymphocyte ratio; APRI, aminotransferase-to-platelet ratio index; ANRI, aspartate aminotransferase-to-neutrophil ratio index; SII, systemic immune-inflammatory index; SIRI, systemic inflammatory response index; FAR, fibrinogen-to-albumin ratio; FLR, fibrinogen-to-lymphocyte ratio; GPR, γ-glutamyl transpeptidase-to-platelet ratio; MELD, end-stage liver disease; ALBI, Albumin-Bilirubin Grade; PNI, prognostic nutritional index.

### Construction of the Novel Blood Index Signature

Univariate Cox analyses were performed to filter blood indices associated with the RFS of HBV-HCC. Blood indices significantly associated with RFS were further analyzed using stepwise multivariate COX regression. Subsequent stepwise elimination of the blood indices resulted in APRI and SIRI as significant predictors for the RFS of HBV-HCC (**Table 2**). The following formula was used to calculate the BIS: 0.447 * Exp(0.340552 * APRI + 0.447854 * SIRI).

**Table 2:** Cox regression of blood indexes for the RFS of HBV-HCC patients.

Variables	Univariate Cox		Stepwise multivariable Cox		
	HR (95% CI)	*p*-Value	HR (95% CI)	Coef	*p*-Value
NLR	1.374 (1.207–1.564)	<0.001			
PLR	1.002 (0.999–1.004)	0.090			
MLR	3.862 (1.720–8.673)	0.001			
FAR	0.935 (0.584–1.496)	0.779			
GPR	1.188 (0.971–1.455)	0.095			
APRI	1.334 (1.118–1.592)	0.001	1.406 (1.201–1.646)	0.340552	<0.001
PNI	0.977 (0.946–1.008)	0.138			
ALBI	1.336 (0.945–1.908)	0.112			
FLR	0.999 (0.982–1.016)	0.922			
SII	1.001 (1.000–1.001)	<0.001			
SIRI	1.507 (1.292–1.757)	<0.001	1.565 (1.346–1.820)	0.447854	<0.001
ANRI	1.010 (1.001–1.019)	0.029			
MELD	0.957 (0.609–1.504)	0.848			

RFS, recurrence-free survival; HBV-HCC, hepatitis B virus-associated hepatocellular carcinoma; HR, hazard ratio; NLR, neutrophil-to-lymphocyte ratio; PLR, platelet-to-lymphocyte ratio; MLR, monocyte-to-lymphocyte ratio; APRI, aminotransferase-to-platelet ratio index; ANRI, aspartate aminotransferase-to-neutrophil ratio index; SII, systemic immune-inflammatory index, SIRI, systemic inflammatory response index, FAR, fibrinogen-to-albumin ratio; FLR, fibrinogen-to-lymphocyte ratio; GPR, γ-glutamyl transpeptidase-to-platelet ratio; MELD, end-stage liver disease; ALBI, Albumin-Bilirubin Grade; PNI, prognostic nutritional index.

### Blood Index Signature and Clinicopathological Parameters

**Figure 1:**
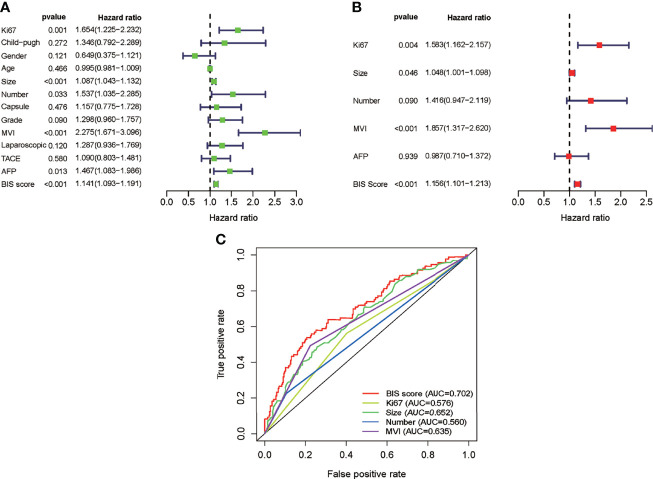
**(A)** Univariate Cox analysis of the blood index signature (BIS) and other clinicopathological characteristics. **(B)** Multivariate Cox analysis of BIS and other clinicopathological characteristics. **(C)** Receiver operating characteristic (ROC) of BIS score and other clinicopathological characteristics.

**Figure 2:**
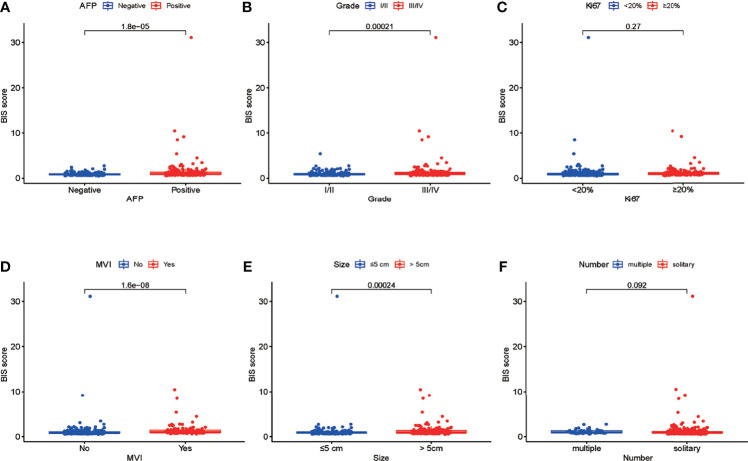
**(A–F)** Differences in the blood index signature (BIS) score between patients with different subgroups of serum alpha-fetoprotein (AFP), tumor grade, Ki67, microvascular invasion (MVI), tumor size, and tumor number.

Univariate Cox regression analysis was used to identify the predicted values of RFS in BIS and clinicopathological parameters. Furthermore, the association between RFS and Ki67, size, tumor number, AFP, MVI, and BIS score was analyzed using (**Figure 1A**) multivariate regression analyses, wherein Ki67, size, MVI, and BIS were found to be independent prognostic predictors of RFS (**Figure 1B**). Moreover, the BIS achieved the highest AUC value as compared with the other clinicopathological parameters (**Figure 1C**). Following this, BIS levels in different clinicopathological subgroups were assessed. The results indicated BIS levels were significantly higher in patients with AFP positive values, grade III/IV, MVI, and tumor size ≥5 cm (p < 0.001) (**Figures 2A–F**).

### Construction of a Nomogram to Predict the Recurrence-Free Survival of Hepatocellular Carcinoma

**Figure 3:**
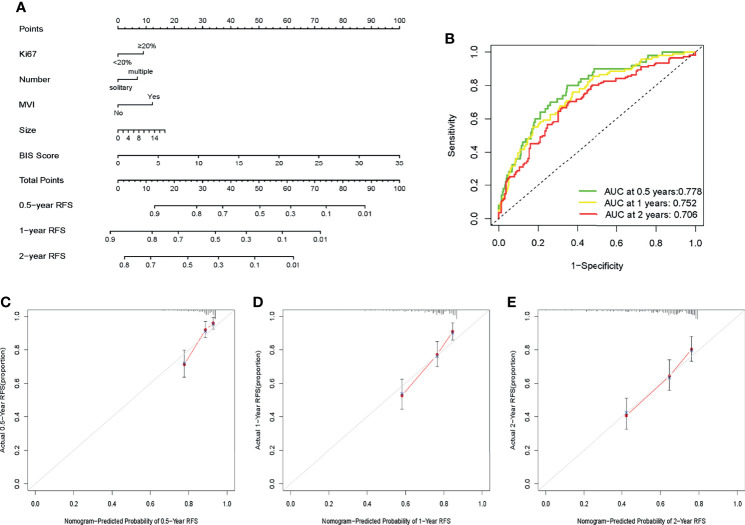
**(A)** Nomogram for predicting the recurrence-free survival (RFS) in patients with hepatitis B virus-associated hepatocellular carcinoma (HBV-HCC). **(B)** Receiver operating characteristic (ROC) curves of the nomogram to predict 0.5-, 1-, and 2-year recurrence-free survival (RFS). **(C–E)** Calibration plots of the nomogram to predict 0.5-, 1-, and 2-year RFS.

In multivariate Cox regression analysis, tumor number with p < 0.1 and hazard ratio (HR) = 1.416 (95% CI = 0.947−2.119) was screened for constructing the model owing to its greater clinical significance. A nomogram was constructed based on Ki67, tumor size, tumor number, MVI, and BIS (**Figure 3A**), with each clinicopathological characteristic corresponding to a specific point. The sum of the values was represented on the total points axis, whereas the probabilities of 0.5-, 1-, and 2-year RFS were represented on the corresponding axis.

### Performance and Clinical Usefulness of the Nomogram

**Figure 4:**
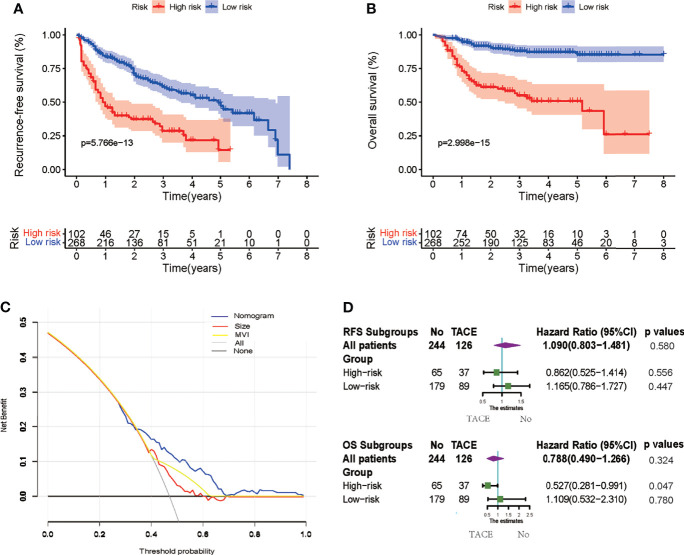
**(A, B)** Kaplan–Meier survival curves to estimate recurrence-free survival (RFS) and overall survival (OS) stratified by risk subgroups. **(C)** Decision curve analysis (DCA) showing the net benefit of the nomogram, microvascular invasion (MVI), and tumor size. **(D)** Subgroup Cox analysis demonstrating the impact of adjuvant transcatheter arterial chemoembolization (TACE) on RFS and OS in different risk subgroups.

The AUC values of ROC of the nomogram were 0.778, 0.752, and 0.706 in 0.5-, 1-, and 2-year RFS prediction, respectively (**Figure 3B**). The calibration plots for the probabilities of 0.5-, 1-, and 2-year RFS demonstrated positive concurrences between the nomogram predictions and actual observations (**Figures 3C–E**). The study cohort was divided into high and low recurrence risk groups according to an optimal cutoff value (28.7) determined using the X-tile software. As shown in **Figures 4A, B**, patients in the low recurrence risk group showed better RFS (p = 5.766e−13) and OS (p = 2.998e−15) than those in the high recurrence risk group. The nomogram showed a better net benefit than that of MVI and tumor size within a wide range of threshold probability (**Figure 4C**).

### Adjuvant Transcatheter Arterial Chemoembolization Efficiency in Different Subgroups

**Figure 5:**
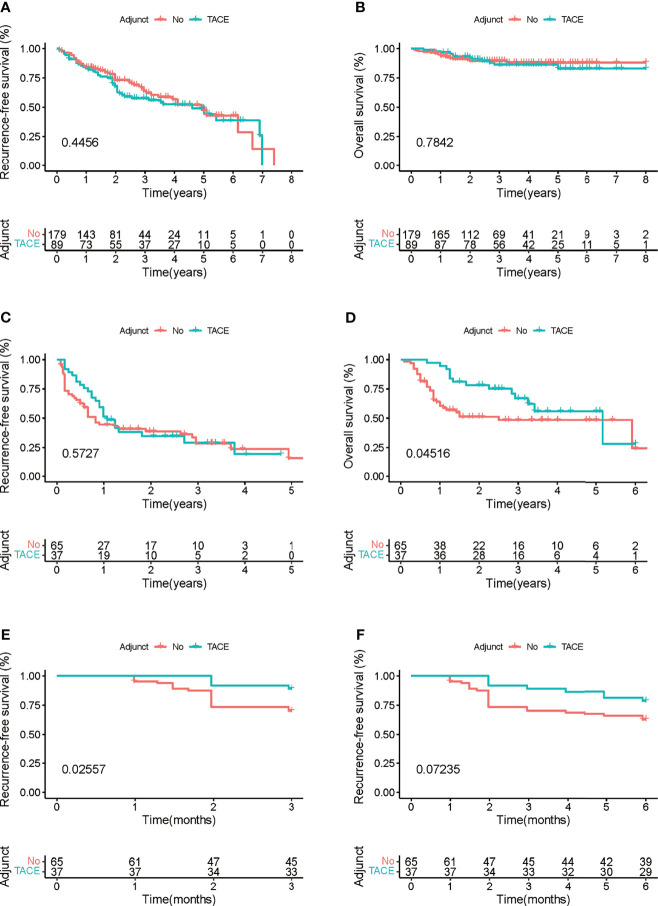
**(A, B)** Kaplan–Meier survival curves of the impact of adjuvant transcatheter arterial chemoembolization (TACE) on recurrence-free survival (RFS) and overall survival (OS) in patients with low recurrence risk. **(C, D)** Kaplan–Meier survival curves of the impact of adjuvant TACE on RFS and OS in patients with high recurrence risk. **(E, F)** Kaplan–Meier survival curves of the impact of adjuvant TACE on RFS and OS in patients with high recurrence risk within 3 and 6 months.

Cox regression analysis was used to determine the efficiency of adjuvant TACE in different subgroups. Patients in the low recurrence risk group, who received adjuvant TACE, showed no improvement in RFS (HR = 1.165, 95% CI = 0.786−1.727, p = 0.447) and OS (HR = 1.109, 95% CI = 0.532−2.310, p = 0.780). However, patients in high recurrence risk group showed improvement in OS (HR = 0.527, 95% CI = 0.281−0.991, p = 0.047) but not in RFS (HR = 0.862, 95% CI = 0.525−1.414, p = 0.556) (**Figure 4D**). Furthermore, the Kaplan–Meier analysis was performed to evaluate the RFS and OS of patients with or without adjuvant TACE in high recurrence and low recurrence risk groups. In the low recurrence risk group, patients who underwent adjuvant TACE showed no benefits regarding RFS (p = 0.4456) and OS (p = 0.7842) (**Figures 5A, B**). Although the patients in the high recurrence risk group who received adjuvant TACE showed no benefits regarding RFS (p = 0.5727), the OS (p = 0.04516) of those who underwent TACE was more beneficial than of those without adjuvant TACE (**Figures 5C, D**). Considering the short-term recurrence rate in the high recurrence risk group, the 3-month recurrence rate was significantly lower in patients who underwent adjuvant TACE (p = 0.02557) (**Figure 5E**). Although the 6-month RFS showed no significant difference, a better and beneficial RFS trend was observed in patients who underwent adjuvant TACE (**Figure 5F**).

## Discussion

In the retrospective study, we constructed an accurate and user-friendly model based on BIS, MVI, Ki67, tumor size, and tumor number to predict the recurrence of HBV-HCC after curative hepatectomy. This model showed good efficacy in discriminating between high and low recurrence risk groups. Notable, adjuvant TACE for patients in the high recurrence risk group showed better OS.

The high rate of recurrence after curative resection remains a great challenge in HCC treatment (21). Previous studies have confirmed that MVI is a critical determinant of the early recurrence of HCC (22, 23). Moreover, Bai et al. demonstrated that Ki67 expression was positively correlated with the increased risk of death and recurrence (24). Additionally, various studies have indicated that systemic inflammation plays a central role in tumor promotion and progression, thereby promoting HCC recurrence postoperatively (25, 26). APRI, a strong indicator of liver inflammation and necrosis in cirrhotic liver, was considered as a risk factor for HCC recurrence postoperatively (27). SIRI was also considered as a reliable marker for prognostic prediction in patients with HCC, which correlated with liver function (28). This study comprehensively analyzed 13 immune indices and confirmed that APRI and SIRI are independent prognostic indices for the recurrence of HBV-HCC after curative hepatectomy. Therefore, an IBS signature was constructed based on APRI and SIRI, which were highly accurate and independent prognostic predictors for recurrence.

However, as adjuvant TACE does not benefit all patients, it is vital to define prognostic categories and recommend TACE to patients who may benefit from this treatment (8, 29). This study provides a novel grouping method based on BIS, which screens patients who benefit from adjuvant TACE. Patients in the low recurrence risk who received adjuvant TACE showed no improvement in RFS and OS. However, patients in the high recurrence risk group who underwent adjuvant TACE showed improved and significantly longer OS but showed no improvement in RFS. The poor OS could be attributed to the high short-term recurrence rate in patients who did not receive TACE. Previous studies have also indicated that early recurrence is associated with worse postoperative survival among patients with HBV-HCC (30–32).

Currently, immunotherapy, especially immune-checkpoint inhibitor therapies, has been successful in treating advanced HCC (33, 34). Combining locoregional therapy approaches such as TACE with immunotherapy is an interesting treatment plan, which may provide better results (35). The BIS model can divide patients into high and low recurrence risk groups, which can be used to filter patients for receiving adjuvant therapy.

However, this study still has several limitations. First, this is a retrospective single-center study, which introduces potential selection bias and has relatively limited evidence. However, the strict exclusion criteria in this study reduced the bias. Second, a validation cohort could not be set up because of the limited case number from a single institution. A validation cohort with a large population can be used in future studies. Third, the efficacy of adjuvant TACE requires further verification using a prospective randomized controlled study, which can help in constructing a more convincing prognostic model for clinical guidance.

## Conclusion

In summary, this study identified and constructed a blood index-based model to predict the HBV-HCC recurrence after curative hepatectomy. This novel model is an effective tool for identifying patients with a high risk of recurrence and who may benefit from adjuvant TACE.

## Data Availability Statement

The raw data supporting the conclusions of this article will be made available by the corresponding author, without undue reservation.

## Ethics Statement

The studies involving human participants were reviewed and approved by The Ethics Committee of Guangdong Provincial People’s Hospital (KYZ202132501). The patients/participants provided their written informed consent to participate in this study.

## Author Contributions

YIZ: collection acquisition, data analyses, and manuscript writing. ZHC, QL, and HH: analyses and interpretation. YUZ, ZRC, and ZM: data acquisition. NS and HJ: project development and critical revisions.

## Funding

This study was supported by the Matching start-up fund of the Natural Science Foundation of China (8200110843), the start-up funding for graduate research projects (JFYS201230034, JFYS201230121), and the Guangdong Medical Science and Technology Research Fund (A2018128).

## Conflict of Interest

The authors declare that the research was conducted in the absence of any commercial or financial relationships that could be construed as a potential conflict of interest.

## Publisher’s Note

All claims expressed in this article are solely those of the authors and do not necessarily represent those of their affiliated organizations, or those of the publisher, the editors and the reviewers. Any product that may be evaluated in this article, or claim that may be made by its manufacturer, is not guaranteed or endorsed by the publisher.
